# Evidence of the Role of R-Spondin 1 and Its Receptor Lgr4 in the Transmission of Mechanical Stimuli to Biological Signals for Bone Formation

**DOI:** 10.3390/ijms18030564

**Published:** 2017-03-07

**Authors:** Gui-Xun Shi, Xin-Feng Zheng, Chao Zhu, Bo Li, Yu-Ren Wang, Sheng-Dan Jiang, Lei-Sheng Jiang

**Affiliations:** Department of Orthopedic Surgery, Xinhua Hospital, Shanghai Jiaotong University School of Medicine, Shanghai 200092, China; shiguixun@163.com (G.-X.S.); zxf272@126.com (X.-F.Z.); chaozhu007@126.com (C.Z.); libo6275819@126.com (B.L.); 13564869748@163.com (Y.-R.W.); jiangsd@126.com (S.-D.J.)

**Keywords:** R-spondin 1, Lgr4, bone formation, bone mechanotransduction, osteoporosis

## Abstract

The bone can adjust its mass and architecture to mechanical stimuli via a series of molecular cascades, which have been not yet fully elucidated. Emerging evidence indicated that R-spondins (Rspos), a family of secreted agonists of the Wnt/β-catenin signaling pathway, had important roles in osteoblastic differentiation and bone formation. However, the role of Rspo proteins in mechanical loading-influenced bone metabolism has never been investigated. In this study, we found that Rspo1 was a mechanosensitive protein for bone formation. Continuous cyclic mechanical stretch (CMS) upregulated the expression of Rspo1 in mouse bone marrow mesenchymal stem cells (BMSCs), while the expression of Rspo1 in BMSCs in vivo was downregulated in the bones of a mechanical unloading mouse model (tail suspension (TS)). On the other hand, Rspo1 could promote osteogenesis of BMSCs under CMS through activating the Wnt/β-catenin signaling pathway and could rescue the bone loss induced by mechanical unloading in the TS mice. Specifically, our results suggested that Rspo1 and its receptor of leucine-rich repeat containing G-protein-coupled receptor 4 (Lgr4) should be a novel molecular signal in the transmission of mechanical stimuli to biological signal in the bone, and this signal should be in the upstream of Wnt/β-catenin signaling for bone formation. Rspo1/Lgr4 could be a new potential target for the prevention and treatment of disuse osteoporosis in the future.

## 1. Introduction

Mechanical stimulus is essential for normal metabolism and functional maintenance of bone. Meanwhile, the skeleton can also respond and adapt to changes of its mechanical environment and maintain a functional homeostasis of bone mass. However, decreased mechanical stimuli on bone for a period of time often lead to disuse osteoporosis and increase the risk for fractures [1,2,3,4]. Maintaining a steady state of bone mass depends on the balance between bone formation and bone resorption, and disruption of this balance under decreased mechanical stimuli is the fundamental mechanism for the bone loss in disuse osteoporosis [1,2,5,6,7]. However, the underlying molecular mechanism for the transmission of mechanical stimuli to biological signals in bone remains poorly understood. Available studies on mechanosensor cells in bone for mechanical response and transduction were mostly focused on osteocytes [8,9,10,11]. Bone marrow mesenchymal stem cells (BMSCs) or mesenchymal progenitor cells (MPCs) and osteoblasts were also reported to be mechanosensitive cells in the skeleton and involved in mechanical stimulation-influenced bone metabolism [12,13,14,15]. In addition, the MAPKs, Wnt/β-catenin and ERK (extracellular signal-regulated kinases) signaling pathways were mostly suggested to be involved in the downstream of the mechanotransduction in the bone [16,17,18]. Nevertheless, there is limited knowledge on the key molecules in the upstream of the transduction.

R-spondins (Rspos) are a family of secreted agonists of the Wnt/β-catenin signaling pathway, and leucine-rich repeat containing G-protein-coupled receptor 4, 5 and 6 (Lgr4/5/6) function as their receptors in the activation of the pathway [19,20,21]. They were widely expressed in developing bone tissues [22] and were recently identified as novel regulators in the skeleton for their positive roles in osteoblastogenesis and bone formation [23,24,25]. Rspo1, a typical member of the family, was able to promote osteoblastic differentiation and bone formation, protect against inflammatory bone damage and attenuate age-related bone loss [26,27,28,29]. However, its exact role and underlying molecular mechanism in mechanical loading-influenced bone metabolism has not been fully understood. It was recently reported that Rspo1 was upregulated by vibration mechanical signals in MPCs and, thus, regarded as a vibration-induced bone-enhancing gene in the skeleton [28]. In addition, the mRNA expression level of Rspo1 was found increased after osteoblastic induction in human primary osteoblasts and FOB1.19 cells [29]. However, is Rspo1 really able to recognize and respond to mechanical stimuli? What is the potential role of Rspo1 or Rspo1-mediated cell signaling in bone mechanotransduction and mechanical loading-influenced bone metabolism? Are the receptors Lgr4/5/6 essential for Rspo1 to work through the Wnt/β-catenin signaling pathway in the promotion of osteoblastic differentiation and bone formation? All of these questions have not been well determined and, therefore, need to be deeply investigated and elucidated.

Thus, in the present study, we determined the sensitivity and response of Rspo1 to mechanical loading changes in BMSCs in vitro and in mice in vivo. Furthermore, the role of Rspo1 and the involved pathway for the transmission of mechanical stimuli to biological signals of bone formation and resorption were also investigated. We hypothesized that Rspo1 should be a mechanosensitive protein and that mechanical stimuli would promote the expression of Rspo1 and subsequent bone formation through the Wnt/β-catenin signaling pathway.

## 2. Results

### 2.1. Mechanical Loading Upregulated the Expression of Rspo1 In Vitro

**Figure 1:**
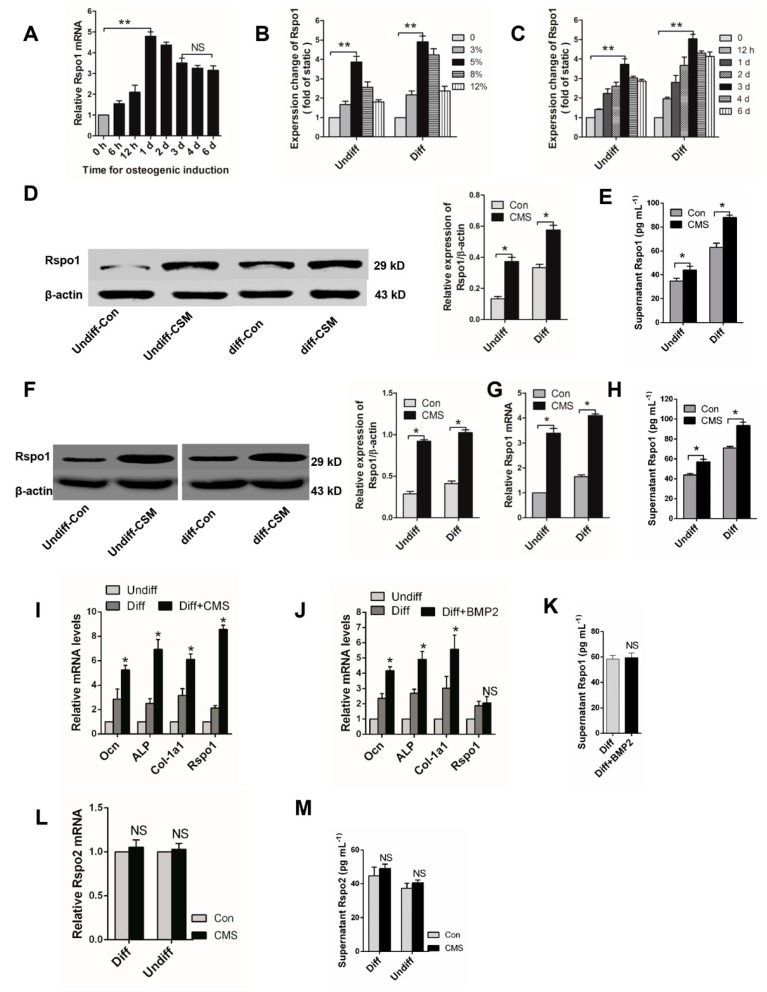
Cyclic mechanical stretch could upregulate the expression of Rspo1 in BMSCs and MC3T3-E1 cells. (**A**) qRT-PCR analysis of Rspo1 in BMSCs during osteogenic differentiation; (**B**) qRT-PCR analysis of Rspo1 in BMSCs treated with cyclic mechanical stretch (CMS) at different elongation (representative illustration for three days after CMS) and (**C**) for different time periods (representative illustration for CMS at 5% elongation); (**D**) Western blot analysis and (**E**) ELISA analysis of Rspo1 in BMSCs treated with CMS (at 5% elongation for three days); (**F**) Western blot analysis, (**G**) qRT-PCR analysis and (**H**) ELISA analysis of Rspo1 in MC3T3-E1 cells treated with CMS (at 5% elongation for three days); (**I**) qRT-PCR analysis of osteogenic differentiation marker genes (*Ocn*, *ALP*, *Col-1a1*) and Rspo1 in BMSCs under CMS (5% elongation) or (**J**) treated with BMP2 for three days; (**K**) ELISA measurement for supernatant Rspo1 in BMSCs treated with BMP2 for three days; (**L**) qRT-PCR analysis and (**M**) supernatant level of Rspo2 in BMSCs treated with CMS (at 5% elongation for three days). Data are presented as the mean ± SD, * *p* < 0.05, ** *p* < 0.01. *p*-Values in (**A**–**C**,**I**,**J**) are based on ANOVA and post hoc analysis, and the other *p*-values were based on Student’s *t*-test. All results are representative of at least three independent experiments. Diff: osteogenic differentiated; Undiff: undifferentiated; Con: static control; CMS: cyclic mechanic stretch; NS: not significant; BMP2: bone morphogenetic protein 2; ELISA: enzyme linked immunosorbent assay.

At first, we examined the dynamic expression of Rspo1 in BMSCs during osteogenic differentiation. qRT-PCR analysis demonstrated that the mRNA level of Rspo1 in the BMSCs quickly increased after osteogenic induction and reached the highest peak after 24 h, then gradually reduced to a relatively steady level on the third day (Figure 1A). To determine the response of Rspo1 to mechanical stimuli, we delivered cyclic mechanical stretching (CMS) at different amounts of elongation (3%, 5%, 8%, 12%) for different time periods (12 h, 1 day, 2 days, 3 days, 4 days, 6 days) to undifferentiated and osteogenic differentiating BMSCs. We found that continuous CMS always led to significant increased mRNA expression of Rspo1 in both undifferentiated and osteogenic differentiating BMSCs. Interestingly, the highest mRNA level of Rspo1 was always found in the cells under CMS at 5% elongation (Figure 1B) or in the cells under CMS for three days (Figure 1C). Thus, we loaded the BMSCs at 5% elongation for three days in our subsequent experiments.

As expected, Western blot analysis demonstrated a significant increase of Rspo1 protein in both undifferentiated and osteogenic differentiating BMSCs under CMS loading at 5% for three days (Figure 1D). Considering that Rspo1 is a secreted protein [30,31], we also measured the supernatant levels of Rspo1. A significantly higher concentration of Rspo1 was found in the supernatants of CMS-loaded BMSCs (both undifferentiated and osteogenic differentiating) than in the static Con (control) group cells (Figure 1E). Furthermore, we verified the effect of CMS on Rspo1 expression and secretion in another pre-osteoblast cell line MC3T3-E1. Results showed that the protein level (Figure 1F), mRNA level (Figure 1G) and supernatant level (Figure 1H) of Rspo1 were all significantly upregulated in MC3T3-E1 cells when treated with 5% CMS for three days.

Previous studies reported that CMS could promote osteoblastic differentiation of BMSCs [12,32]. In this study, we found that the osteogenic differentiation marker genes *Ocn*, *Col-1a1* and *ALP* were all upregulated along with Rspo1 in the BMSCs under continuous CMS (Figure 1I). To clarify whether the upregulated Rspo1 resulted from CMS per se or from subsequently enhanced osteogenic differentiation, we also treated the BMSCs with BMP2 (bone morphogenetic protein 2), which was able to promote osteoblastic differentiation and bone formation. After being treated with BMP2 (50 ng/mL) for three days, enhanced osteoblastic activity was found in osteogenic differentiating BMSCs (Figure 1J). However, the mRNA and supernatant level of Rspo1 were not significantly increased in BMP2-treated cells (Figure 1J,K). These results indicated that the upregulated Rspo1 was caused by CMS per se instead of subsequently promoted osteoblastic differentiation. In addition, we also found that Rspo2, another potential modulator of osteoblastic differentiation in the Rspo family [23,33], was not sensitive to CMS stimulation in BMSCs (Figure 1L,M). Considering the lack of information of another two Rspo proteins (Rspo3 and Rspo4) related to bone metabolism [24], we did not investigate the expression of Rspo3 and Rspo4 in BMSCs under CMS.

### 2.2. Mechanical Unloading Downregulated the Expression of Rspo1 in BMSCs In Vivo

**Figure 2:**
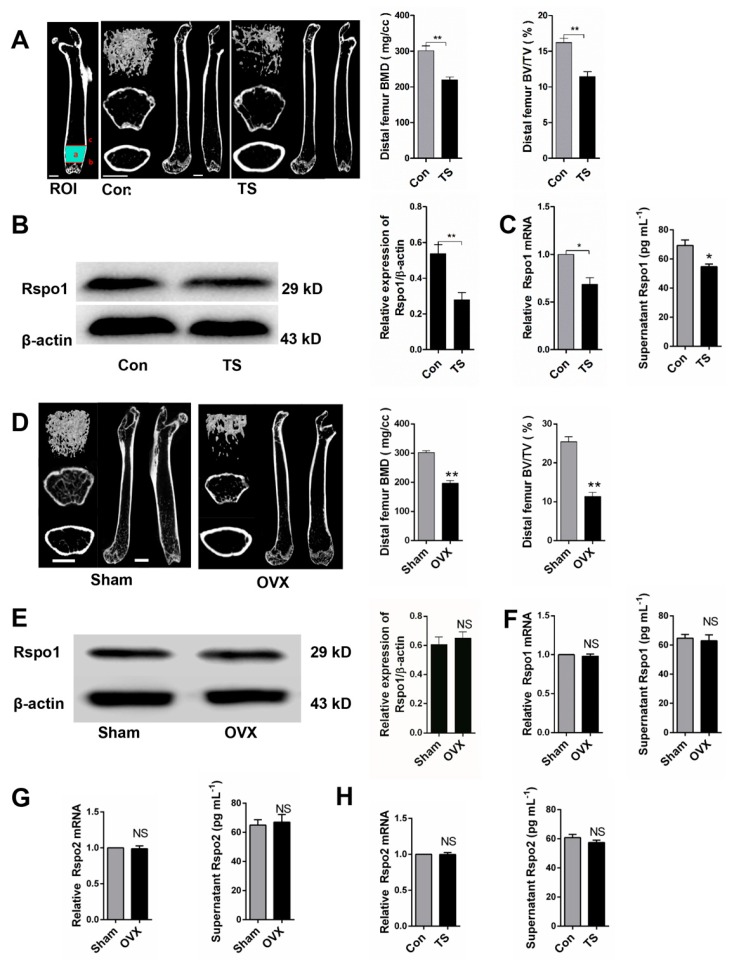
Mechanical unloading resulted in decreased expression of Rspo1 in BMSCs in vivo. (**A**) Representative μCT images and quantification analysis of bone parameters of distal femurs in Con and tail suspension (TS) mice. *n* = 6. Scale bars, 1 mm; (**B**) Western blot analysis and (**C**) mRNA expression and supernatant content analysis of Rspo1 in the BMSCs from TS and Con group mice bones; (**D**) representative μCT images and quantification analysis of bone parameters of distal femurs in sham and OVX mice. *n* = 6. Scale bars, 1 mm; (**E**) Western blot analysis and (**F**) mRNA expression analysis and supernatant content of Rspo1 in the BMSCs from OVX and sham group mice bones; (**G**,**H**) mRNA expression and supernatant content analysis of Rspo2 in the BMSCs from the two group of mice bones. Data are presented as the mean ± SD, * *p* < 0.05, ** *p* < 0.01. All *p*-values are based on Student’s *t*-test. Con: control; TS: tail suspension; Sham: sham surgery; OVX: ovariectomized; µCT: micro-computed tomography; ROI: region of interest.

We successfully constructed a well-established hindlimb unloading mouse model by tail suspension (TS) [34,35] and confirmed that the unloading induced bone loss. μCT analysis of the distal femurs demonstrated significantly decreased bone mineral density (BMD) and bone volume over total volume (BV/TV) in the TS group (Figure 2A). Subsequently, we found significantly decreased Rspo1 in BMSCs from TS bones, which were estimated by Western blot analysis (Figure 2B), qRT-PCR analysis and ELISA (enzyme linked immunosorbent assay) analysis (Figure 2C).

To determine whether the downregulated Rspo1 in vivo was caused by mechanical unloading per se or by subsequent osteoporosis, we established another classical osteoporosis mouse model and evaluated the Rspo1 expression in ovariectomized (OVX) mice. Bone loss and osteoporosis were proved in the OVX mice by μCT analysis (Figure 2D). Interestingly, Western blot analysis (Figure 2E), qRT-PCR analysis and ELISA analysis (Figure 2F) of the BMSCs from femur and tibia bones did not show any significant difference of Rspo1 expression between the OVX and sham groups. These results suggested that the downregulated Rspo1 in BMSCs in vivo probably resulted from unloading per se rather than from the subsequent osteoporosis. However, there also may be sex differences that account for these results due to the lack of unloading female mice or orchidectomy mice model experiments. In addition, we measured the expression of another Rspo protein, Rspo2, in ex vivo BMSCs, and no difference was found between the Con and TS group, as well as between the Sham and OVX group (Figure 2G,H).

### 2.3. Rspo1 Promoted the Osteogenic Differentiation of BMSCs through the Wnt/β-Catenin Signaling Pathway

**Figure 3:**
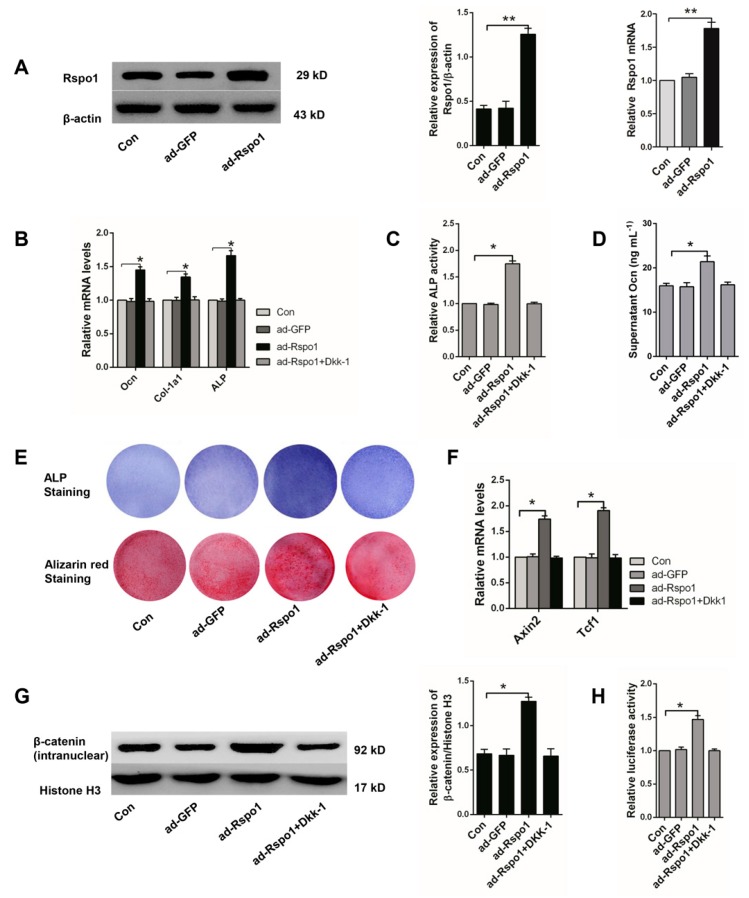
Overexpression of Rspo1 enhanced osteogenic differentiation of BMSCs via the Wnt/β-catenin signaling pathway. (**A**) Western blot and qRT-PCR analysis of Rspo1 after adenovirus infection; (**B**) qRT-PCR analysis of osteogenic differentiation markers *Ocn*, *Col-1a1* and *ALP* (Day 3); (**C**) Relative ALP activities (Day 3); (**D**) ELISA measurement for supernatant Ocn (Day 3); (**E**) Representative images of ALP staining (Day 7) and Alizarin red staining (Day 12); (**F**) qRT-PCR analysis of the Wnt target genes Axin2 and Tcf1; (**G**) Western blot analysis of the intranuclear level of β-catenin and (**H**) Topflash dual-luciferase activity assay of BMSCs after 24 h of osteogenic differentiation. The luciferase activities were presented as fold changes normalized to Renilla compared to Con group cells. All data were confirmed by three repeated tests and presented as the mean ± SD, * *p* < 0.05, ** *p* < 0.01. *p*-Values are based on ANOVA and post hoc analysis. ad-GFP: blank adenovirus with green fluorescent protein; ad-Rspo1: adenovirus with overexpressed Rspo1.

Rspo1 was identified to promote the osteoblastic activity in C2C12, FOB1.19 and MC3T3-E1 cells [27,29]. To investigate the role of Rspo1 in the osteogenic differentiation of BMSCs, we overexpressed it in normal BMSCs by adenovirus infection (Figure 3A) and found that Rspo1 overexpression significantly increased the osteogenic differentiation markers (Ocn, Col-1a1, ALP) (Figure 3B), ALP activities (Figure 3C) and supernatant Ocn levels (Figure 3D) after three days of osteogenic induction. ALP staining and the Alizarin red staining assay showed that Rspo1 overexpression increased ALP activity and mineralized deposits (Figure 3E). All of these results suggested the promotion effect of Rspo1 on the osteogenic differentiation of BMSCs.

As Rspo proteins were recognized as agonists of Wnt/β-catenin signaling, we evaluated the signaling level in the ad-Rspo1 infected BMSCs during osteogenic differentiation. As expected, significantly increased Wnt target genes (Axin2, Tcf1) (Figure 3F), intranuclear β-catenin (Figure 3G) and luciferase activity (Figure 3H) were found, which all indicated the promoted activity of the Wnt/β-catenin signaling in osteogenic-induced BMSCs by Rspo1 overexpression.

To further clarify whether the potentiation effect of Rspo1 on osteogenic differentiation of BMSCs was dependent on the Wnt/β-catenin pathway, we treated the ad-Rspo1 cells with Dkk-1 (100 ng/mL), a blocker of the pathway, and found that the enhanced osteogenic differentiation by Rspo1 overexpression was completely abolished (Figure 3B–E) by the inhibition of the Wnt/β-catenin pathway (Figure 3F–H). This suggested that the potentiation of Rspo1 on the osteogenic differentiation of BMSCs was working through the activation of the Wnt/β-catenin pathway.

### 2.4. Rspo1 Was Essential in CMS-Promoted Osteogenic Differentiation of BMSCs

**Figure 4:**
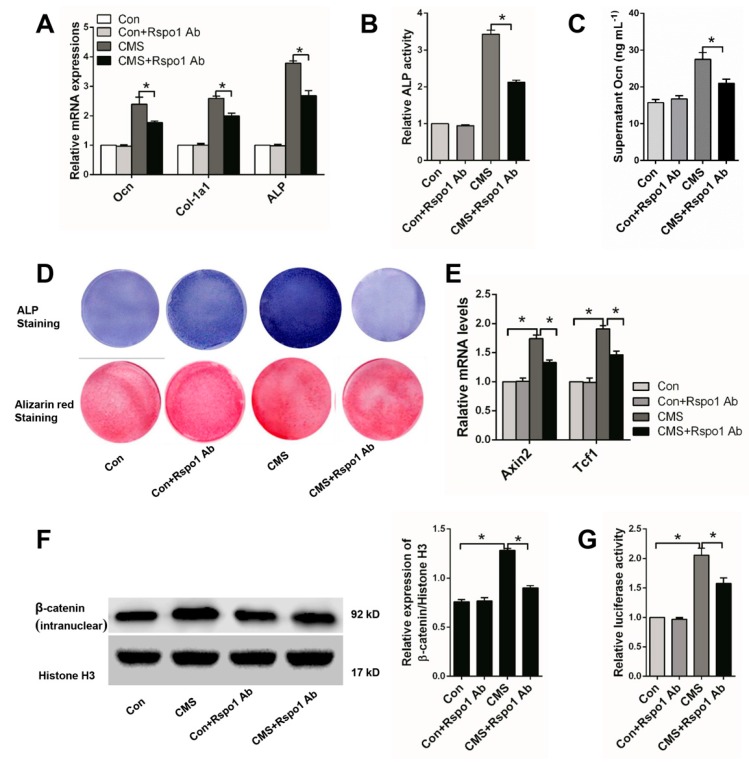
Rspo1 neutralizing antibody (Rspo1 Ab) (1 μg/mL) abolished the enhanced osteogenic differentiation and Wnt/β-catenin activity by CMS (5% elongation) in BMSCs. (**A**) qRT-PCR analysis of osteogenic differentiation markers *Ocn*, *Col-1a1* and *ALP* (Day 3); (**B**) relative ALP activities (Day 7); (**C**) ELISA measurement for supernatant Ocn (Day 3); (**D**) Representative images of ALP staining (Day 7) and Alizarin red staining (Day 14); (**E**) qRT-PCR analysis of the Wnt target genes Axin2 and Tcf1; (**F**) Western blot analysis of the intranuclear level of β-catenin and (**G**) Topflash dual-luciferase activity assay of BMSCs after 24 h of osteogenic differentiation. The luciferase activities were presented as fold changes normalized to Renilla compared to Con group cells. All data were confirmed by three repeated tests and presented as the mean ± SD, * *p* < 0.05. *p*-Values are based on ANOVA and post hoc analysis.

It was well known that mechanical stimulation could promote the osteogenic differentiation of BMSCs [12,15,32,36]. We also found significantly enhanced osteogenic differentiation of the BMSCs after being treated with 5% CMS for three days (Figure 1I). To further clarify the relationship between CMS-upregulated Rspo1 and CMS enhanced osteogenic differentiation of BMSCs, we respectively added Rspo1 neutralizing antibody (1 μg/mL) into the osteogenic differentiation medium of Con and CMS group cells. Then, we found that Rspo1 neutralizing antibody had few effect on the osteogenic differentiation of the Con group cells, but it significantly impaired the osteogenic differentiation of the CSM group cells (Figure 4A–D).

Considering the key role of the Wnt/β-catenin signaling pathway in mechanical stimulation-influenced osteoblastic differentiation and bone formation [11,18,32,37,38], we also investigated the signaling activity in Con and CMS cells treated with or without Rspo1 neutralizing antibody. We found that 5% CMS did enhance the Wnt/β-catenin signaling activity, and the Rspo1 neutralizing antibody significantly weakened the signaling activity in the CMS group cells (Figure 4E–G).

The above results indicated that CMS-upregulated Rspo1 in BMSCs led to the subsequent potentiated Wnt/β-catenin activity and osteogenic differentiation of BMSCs. Therefore, we suggested that Rspo1 should be an important link in the transmission of mechanical stimuli to biological signals for bone formation.

### 2.5. Adenovirus-Mediated Delivery of Rspo1 Increased the Bone Mass of TS Mice

**Figure 5:**
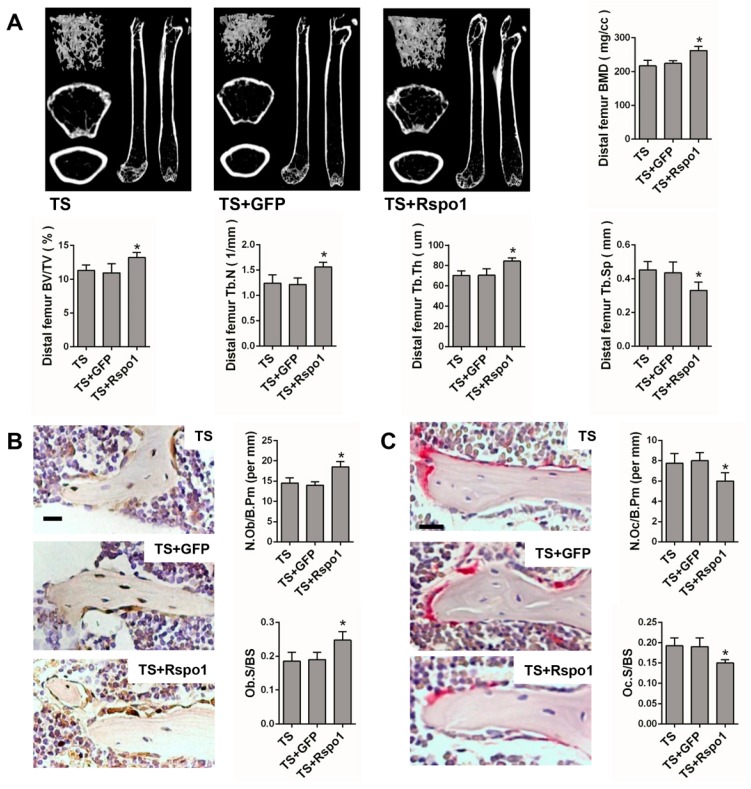

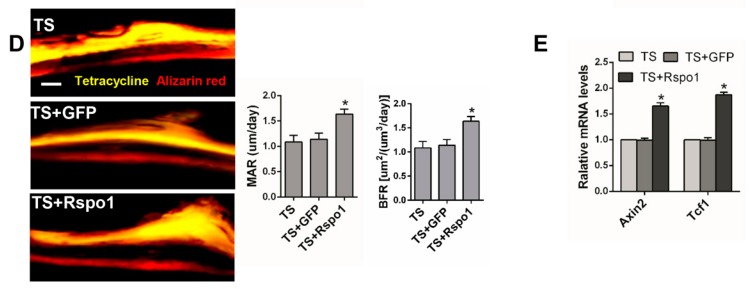
The bone mass and bone formation were significantly increased in the TS mice model by treating with Rspo1. (**A**) Representative μCT images and quantification analysis of bone parameters of distal femurs in TS, TS + GFP and TS + Rspo1 mice. *n* = 6. Scale bars, 1 mm; (**B**) Representative images of immunostaining with antibody to OPG and morphometric analysis of osteoblasts surrounding trabecular bones of distal femurs. *n* = 6. Scale bar, 40 μm; (**C**) Representative Trap staining images and morphometric analysis of osteoclasts surrounding trabecular bones of distal femurs. *n* = 6. Scale bar, 30 μm; (**D**) Representative images and quantitative analysis of new bone formation assessed by tetracycline and alizarin red fluorescence double-labeling in each group. Scale bar, 20 μm. *n* = 4; (**E**) qRT-PCR analysis of the Wnt target genes Axin2 and Tcf1 in distal femurs of each group. *n* = 4. Data are presented as the mean ± SD, * *p* < 0.05. All *p*-values are based on ANOVA and post hoc analysis. MAR, mineral apposition rate; BFR, bone formation rate; BMD, bone mineral density; BV/TV, bone volume over total volume; Tb.Th, trabecular thickness; Tb.N, trabecular number; Tb.Sp, trabecular separation; Ob.S/BS: osteoblast surface per bone surface; N.Ob/B.Pm: number of osteoblasts per bone parameter; Oc.S/B.S: osteoclast surface per bone surface; N.Oc/B.Pm: number of osteoclasts per bone parameter.

To confirm the in vivo function of Rspo1 on mechanical unloading induced bone loss, we examined the effect of adenovirus-mediated delivery of Rspo1 (ad-Rspo1) in a TS mice model of unloading-induced osteoporosis (Figure 2A). When Rspo1 was delivered, the distal femur bone mass, BMD and trabecular parameters in TS mice were significantly improved, shown by μCT analysis, whereas ad-GFP did not show a similar effect (Figure 5A). Meanwhile, application of ad-Rspo1 in the TS mice led to significantly increased bone formation parameters of Ob.S/BS (osteoblast surface per bone surface) and N.Ob/B.Pm (number of osteoblasts per bone parameter) as assessed by immunohistochemistry staining of OPG (osteoprotegrin) (Figure 5B), while the bone resorption parameters of Oc.S/BS (osteoclast surface per bone surface) and N.Oc/B.Pm (number of osteoclasts per bone parameter) as assessed by Trap staining were significantly decreased (Figure 5C). Furthermore, delivery of ad-Rspo1 in the TS mice resulted in significantly increased new bone formation and higher mineral apposition rate (MAR) and bone formation rate (BFR) as demonstrated by bone dynamic histomorphometric analysis (Figure 5D). Besides, we also examined the enhanced Wnt/β-catenin activity by ad-Rspo1 in vivo proven by the increased expression of Axin2 and Tcf1 (Figure 5E). These results demonstrated the effectiveness of Rspo1 in treating unloading-induced bone loss.

### 2.6. Lgr4 Was Essential for Rspo1 to Enhance the Osteogenic Differentiation and Wnt/β-Catenin Signaling of BMSCs

**Figure 6:**
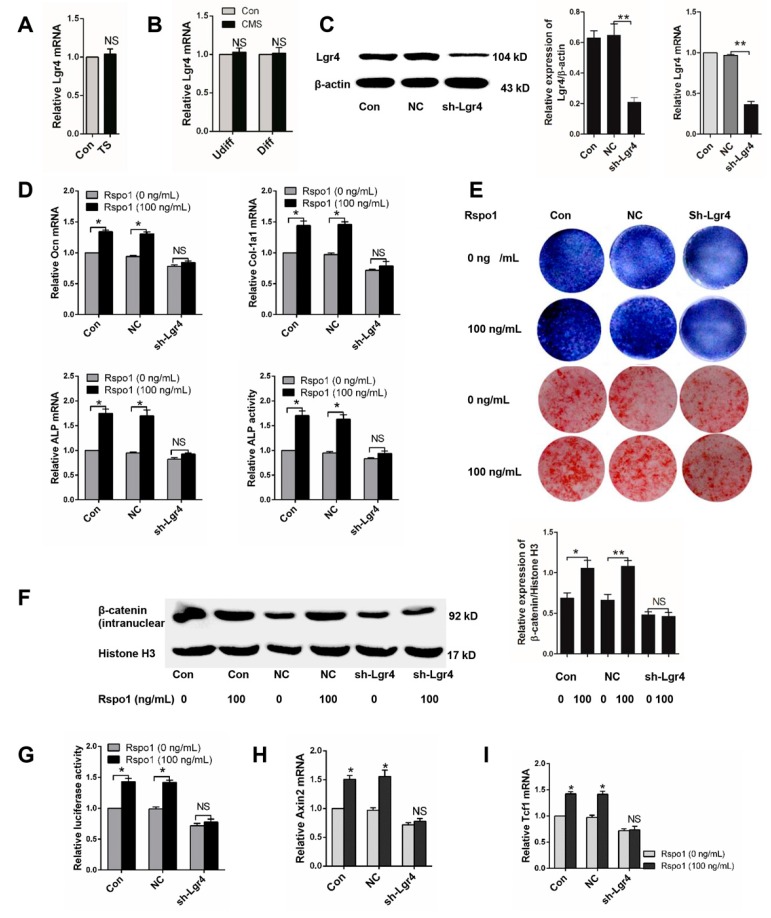
Lgr4 was essential for Rspo1 to promote osteogenic differentiation and Wnt/β-catenin signaling activity in BMSCs. (**A**) qRT-PCR analysis of Lgr4 in distal femurs of Con and TS mice (*n* = 6) and (**B**) in BMSCs treated with 5% CMS; (**C**) Western blot and qRT- PCR analysis of Lgr4 after lentivirus sh-Lgr4 infection; (**D**) qRT-PCR analysis of osteogenic differentiation markers (*Ocn*, *Col-1a1*, *ALP*) and ALP activities assay of lentivirus infected BMSCs after three days of osteogenic induction with or without Rspo1 (100 ng/mL); (**E**) representative images of ALP staining (Day 10) and Alizarin red staining (Day 18) of each group cells; (**F**) Western blot analysis of the intranuclear level of β-catenin and (**G**) Topflash dual-luciferase activity assay of BMSCs after 24 h of osteogenic differentiation; (**H**) qRT-PCR analysis of the Wnt target genes Axin2 and (**I**) Tcf1. All data were confirmed by three repeated tests and are presented as the mean ± SD, * *p* < 0.05, ** *p* < 0.01. All *p*-values are based on Student’s *t*-test. NC: negative control; sh-Lgr4: short hairpin RNA-Lgr4

Lgr4 was identified as one of the receptors of Rspo1 [21,39] and suggested to be crucial in regulating bone development and metabolism [33,40,41]. Therefore, we wondered if the role of Lgr4 in Rspo1 promoted osteogenic differentiation of BMSCs. To better understand the Rspo1 signal in mechanical loading-influenced osteogenesis based on the above research, we firstly examined the expression of Lgr4 under mechanical unloading in vivo or CMS in vitro and found that mechanical unloading or CMS had no impact on Lgr4 expression (Figure 6A,B). Next, we silenced Lgr4 in BMSCs using lentivirus-mediated shRNA infection (sh-Lgr4 group), and the blank lentiviral vector carrying GFP-infected cells were set as the negative control (NC group). Successful knockdown of Lgr4 was verified by Western blot and qRT-PCR analysis (Figure 6C). Then, we treated the BMSCs with or without recombinant Rspo1 (100 ng/mL) during osteogenic differentiation and assessed the influence of Rspo1 on osteogenic differentiation in each group. Unlike those in the non-silenced cells or negative control cells, the mRNA expression of osteoblastic marker genes (*Ocn*, *ALP*, *Col-1a1*) and the ALP activity (Figure 6D), the ALP staining and osteogenic mineralization (Figure 6E) in the sh-Lgr4 group cells did not increase significantly in the presence of Rspo1. Meanwhile, no remarkable increase of intranuclear β-catenin (Figure 6F), or luciferase activity (Figure 6G), or Axin2 (Figure 6H), or Tcf1 (Figure 6I) was detected in the Lgr4-silenced cells. These results suggested that Lgr4 should be essential for Rspo1 to potentiate osteoblastic differentiation and Wnt/β-catenin signaling in BMSCs.

## 3. Discussion

The ability of the skeleton to respond and adapt to mechanical stimuli is critical for the maintenance of its function and bone mass. The response and adaptation of the skeleton to mechanical stimuli is dependent on the healthy and efficient mechanotransduction involving many unclarified molecular signals in the bone [16,42]. In this study, we found that Rspo1 was a mechanosensitive protein for bone formation. The expression of Rspo1 increased in BMSCs and MC3T3-E1 cells under CMS in vitro and decreased in the unloading bones in vivo. On the other hand, Rspo1 could promote the osteogenesis of BMSCs under CMS through activating the Wnt/β-catenin signaling pathway and could alleviate the unloading-induced bone loss in mice. Specifically, our results suggested that Rspo1/Lgr4 should be a novel molecular signal for the transmission of mechanical stimuli to biological signals in the bone, and this signal should be in the upstream of Wnt/β-catenin signaling for bone formation.

Rspos were known as a family of secreted agonists of the Wnt/β-catenin signaling pathway and were recently identified as a key modulator in skeleton development and postnatal bone remodeling [23,24]. However, how the expression or secretion of Rspos was regulated in bone or bone cells was still poorly understood [31]. It was recently reported that Rspo1 was upregulated by vibration mechanical signals in MPCs [28], and the mRNA expression of Rspo1 was increased after osteoblastic induction in human primary osteoblasts and FOB1.19 cells [29]. Our in vitro study demonstrated that the expression of Rspo1 increased in BMSCs during osteogenic differentiation, and CMS could upregulate the expression of Rspo1 in both undifferentiated and osteogenic differentiating BMSCs. Besides, the upregulated expression of Rspo1 by mechanical stimulation was also verified in MC3T3-E1 cells. Our in vivo study showed the decreased expression of Rspo1 in cultured BMSCs from TS bones after unloading for 28 days, but not in the BMSCs from OVX bones for one month, which indicated, to some extent, the possibility that mechanical unloading resulted in the decreased expression of Rspo1. These findings supported our speculation that Rspo1 should be a mechanosensitive protein and might be involved in mechanotransduction in the skeleton. Notably, we also found that CMS increased the supernatant level of Rspo1 in vitro, which indicated that mechanical stimuli could regulate the secretion of Rspo1 in BMSCs or preosteoblasts. However, to certify the mechanosensitivity of Rspo1 in vivo more convincingly, it is better to investigate whether reloading could upregulate the expression of Rspo1 in BMSCs after a period of unloading, which is a shortage of the present study. Previous studies reported that Rspo1 had important roles in the regulation of bone metabolism and remodeling. At first, Rspo1 was found to be able to induce osteoblastic differentiation and bone remodeling synergized with Wnt3A in vitro [26]. Later, Rspo1 was proven to protect against inflammation-induced bone and cartilage damage in vivo by promotion of bone formation and inhibition of bone resorption [27]. More recently, Rspo1 was reported to promote bone formation in three age-related bone loss mouse models [28]. In addition, Rspo1 was suggested to play regulatory roles in osteoblastic differentiation and bone formation through the Wnt/β-catenin signaling pathway [24,26,27,29]. In our present study, we not only confirmed previous reports that Rspo1 could promote osteoblastic differentiation and bone formation, but also found that the receptor Lgr4 was essential for Rspo1 to potentiate osteogenic differentiation through activating the Wnt/β-catenin signaling in the BMSCs.

Activation of the Wnt/β-catenin signaling was reported to be a normal response to the mechanical stimulation in bone [11,18,37,43]. However, the upstream molecular mechanism of this activation of the pathway by mechanical stimulation was incompletely understood. Available studies regarded osteocytes as the primary mechanosensor and emphasized the sclerostin and Wnt/Lrp5 signal in the skeleton to activate the Wnt/β-catenin pathway for bone formation under mechanical stimulation [1,9,38,44,45,46,47]. However, sclerostin should not be the only modulator of the mechanic response in bone, and there could be other factors responsible for sensing, interpreting and transmitting the mechanic signals in the skeleton [48]. In the present research, we confirmed that Rspo1 was another mechanosensitive protein in bone. Moreover, we suggested that Rspo1/Lgr4 should be a novel upstream modulator of the Wnt/β-catenin signaling in bone, which was responsible for the transmission of mechanical stimuli to biological signals for bone formation. These findings provided, at least to some extent, new information on understanding the mechanisms of bone mechanotransduction and unloading-induced bone loss.

Disuse osteoporosis is prevalent in the situations of fracture immobilization, neurological or muscular diseases, spinal cord injury, space flight or long-term bed rest [2,4,49,50,51]. However, in clinical practice, the traditional anti-resorption drugs were sometimes less sensitive to disuse osteoporosis [52]. Our findings provided in vivo validation of the therapeutic effect of Rspo1 on unloading-induced bone loss, which revealed another novel potential target for the development of an anabolic therapeutic agent for disuse osteoporosis.

## 4. Materials and Methods

### 4.1. Animals and Grouping

Thirty-two male C57BL/6J mice (age 6 months, Shanghai SLAC Laboratory Animal Co., Ltd., Shanghai, China) were randomly assigned into four groups (*n* = 8 per group): Con group (normal caged and weight bearing freely as the control), TS group (hindlimb unloading by tail suspension), TS + GFP group (tail suspension and accepted injection of adenovirus blank vector carrying green fluorescent protein (GFP) as negative control) and TS + Rspo1 group (tail suspension and accepted injection of adenovirus carrying a promoter to over-express Rspo1). Another sixteen C57BL/6J female mice (age 6 weeks, Shanghai SLAC Laboratory Animal Co. Ltd., China) were randomly assigned into two groups (*n* = 8 per group): sham operation group (sham) and surgical ovariectomy group (OVX).

All mice were sacrificed by using euthanasia, and bilateral femurs were harvested for the subsequent experiments. All in vivo experiments and subsequent analysis of outcomes were done with blinding to investigators. The animal care procedures were conducted in accordance with the guidelines of our University Committee (Ethics Committee of Xinhua Hospital Affiliated to Shanghai Jiao Tong University School of Medicine, XHEC-F-2017-005, 3 March 2017) on Animal Use and Care.

### 4.2. Hindlimb Unloading and Adenovirus Injection of Mice

Twenty-four mice were subjected to hindlimb unloading by tail suspension according to the previously described methods for a period of 28 days [32,34]. They were housed individually under the same standard conditions (21 °C homoiothermy, 12 h light/dark cycle) as the Con group mice with free access to adequate rodent chow and water. The mice were monitored at least once a day about general physical condition, suspension height, tail blood circulation, and so on. The mice in the TS + GFP group and TS + Rspo1 group accepted adenovirus injection into the bone marrow cavity of bilateral femurs, with a dose of 20-μL virus solution (5 × 10^8^ pfu)/limb by using a 25-μL microsyringe (Hamilton, 1702 RN, Reno, NV, USA) once a week [53,54].

### 4.3. OVX Mice Model

The mice were subjected to bilateral surgical ovariectomy or sham surgery (the ovaries were left intact) under general anesthetization with chloral hydrate. After one month, all mice were sacrificed by euthanasia for subsequent experiments.

### 4.4. Cell Culture and Induction of Osteogenic Differentiation

BMSCs derived from the bone marrow of C57BL/6J mice at passage 6 were purchased from Cyagen Biosciences (Guangzhou, China). Cells were maintained in special C57BL/6 Mouse Mesenchymal Stem Cell growth medium (Cyagen Biosciences), containing 10% fetal bovine serum (FBS), 1% glutamine and 1% penicillin-streptomycin and incubated at 37 °C in a humidified atmosphere with 5% CO_2_. When the cells grew to 80%–90% confluence, they were detached using 0.25% trypsin, resuspended in growth medium and passaged 1:3 onto 10-cm dishes. For induction of osteogenic differentiation, BMSCs were seeded onto six-well plates at a density of 2.0 × 10^5^ cells per well. When the confluence reach to 80%, culture medium were changed with special C57BL/6 Mouse Mesenchymal Stem Cell osteogenic differentiation medium (Cyagen Biosciences) containing 10% FBS, 1% penicillin-streptomycin, 1% glutamine, 0.2% ascorbate, 1% β-glycerophosphate and 0.01% dexamethasone. All BMSCs used in the experiment were at passage 7–10. In a similar way, murine pre-osteoblast MC3T3-E1 cells (American Type Culture Collection, Manassas, VA, USA, ATCC CRL-2593) were cultured in α-modified minimal essential medium (Hyclone, GE Healthcare Life Sciences, Pittsburgh, PA, USA) containing 10% FBS and then osteogenically induced using the BMSC osteogenic differentiation medium. To test the osteogenesis, real-time PCR analysis of the osteoblastic marker genes, ALP activity assay, ALP and Alizarin red staining were performed. The growth or osteogenic differentiation medium was replaced every 3 days.

The BMSCs used in the in vivo experiment were isolated from mice femur and tibia bone marrow after unloading for 28 days or OVX for one month, selected with the continuous adherence method and then cultured for amplification in the same way as above.

### 4.5. Adenovirus or Lentivirus Transfection

Cells were seeded onto six-well plates, and adenovirus solution (carrying GFP or a promoter to over-express Rspo1) was added into the growth medium at an MOI (multiplicity of infection) of 80 to transfect BMSCs after a hard attachment of 12 h. After another 12 h, the medium containing adenovirus was changed with normal growth medium. For lentivirus infection, lentiviral particles expressed GFP or short hairpin RNA interference targeting Lgr4 (sh-Lgr4, 5′-CCGGCAAAGAACAGGTGCCTAAATTCTCGAGAATTTAGGCACCTGTTCTTTGTTTTTG-3′) were added into the growth medium at an MOI of 100. Positive stable transfectants were selected by using puromycin and expanded for the subsequent study. To test transfection efficiency, cells were observed and photographed under a fluorescence microscope after transfection for 48 and 72 h. The efficiency of knockdown or overexpression of target gens was confirmed by real-time PCR and Western blot analysis. Both the recombinant adenovirus and lentivirus vectors were purchased from Hanbio Biotechnology Co., Ltd. (Shanghai, China). Cells without any transfection were used as the control.

### 4.6. CMS Stimulation for BMSCs and MC3T3-E1 Cells

BMSCs and MC3T3-E1 cells were seeded on BioFlex six-well plates, which carried flexible and collagen-I coated silicone rubber membranes (Flexcell International Corporation, Burlington, NC, USA) and subjected to continuous CMS stimuli with a 0.5-Hz sinusoidal waveform after tight attachment for 6–8 h using an FX-5000T Flexercell Tension Plus unit (Flexcell International Corporation). The BMSCs were respectively subjected to continuous CMS at 3%, 5%, 8% or 12% elongation for a constant time of 3 days (experiments at a constant elongation of 5% for 12 h, 1 day, 2 days, 3 days, 4 days and 6 days were also separately performed). The MC3T3-E1 cells were subjected to continuous CMS at 5% elongation for 3 days. The cells grown on BioFlex six-well plates, but without CMS were taken as the control. All BioFlex culture plates were identically incubated in a humidified atmosphere at 37 °C and 5% CO_2_. When CMS procedure finished, cells and supernatants were immediately harvested for further Western blot, ELISA and real-time PCR analysis.

### 4.7. TOPflash Dual-Luciferase Reporter Assays

The adenoviral transient infected or selected stable lentiviral infected BMSCs were plated onto six-well plates with growth medium at a density of 2 × 10^5^ cells/well for 12 h of adherence. Then, cells were co-infected with mixed plasmids containing 500 ng of Topflash/Fopflash firefly and 20 ng of SV-40 Renilla luciferase constructs (Biovector, Inc., Beijing, China) per well for 24 h. The medium was then changed with new growth medium, and subsequently, osteogenic differentiation induction was performed after another 24 h. After induction for 24 h, cell lysates were isolated, and the luciferase activity was measured using the Dual-Luciferase Assay kit (Promega, Beijing, China) according to the manufacturer’s protocol. The luciferase activity was presented as the ratio of TOPflash and FOPflash.

### 4.8. Protein Extraction and Western Blot Analysis

Total protein was isolated from cells using the RIPA (Radio-Immunoprecipitation Assay) lysis buffer. Nucleoprotein from cells was extracted using the Nucleoprotein Extraction Kit (Sangon Biotech, Shanghai, China) according to the manufacturer’s instructions. The concentration of all of the extracted protein samples was measured by the BCA Kit (Beyotime, Shanghai, China) according to the protocol. Protein samples were separated by SDS-PAGE (8% or 10% polyacrylamide gels) and waterishly transferred to polyvinylidene difluoride (PVDF) membranes. We blocked membranes with 5% nonfat milk for 1 h and then incubated overnight at 4 °C with specific primary antibodies against Rspo1 (1:1000 dilution, ab106556), Lgr4 (1:1000 dilution, ab137480), Histone H3 (1:1000 dilution, ab33309) from Abcam (Cambridge, MA, USA) and β-catenin (1:1000 dilution, D10A8), β-actin (1:1000 dilution, 13E5) from Cell Signaling Technology (San Antonio, TX, USA). After rinsing, the membrane was incubated with horseradish peroxidase-conjugated anti-rabbit secondary antibody for 1 h and visualized using the enhanced chemiluminescence detection system (Millipore, Billerica, MA, USA).

### 4.9. RNA Isolation and Quantitative Real-Time PCR

We isolated the total RNA from cells and distal femur bone tissues using TRIzol reagents (Invitrogen, Bartlesville, OK, USA) according to the manufacturer’s instructions. Reverse transcription was performed, and 2-μg cDNA aliquots were synthesized using the PrimeScript RT reagents kit (Takara, Beijing, China) according to the manufacturer’s protocol. We then performed the real-time PCR in a 20-μL reaction system using a SYBR Premix Ex TaqTM kit (Takara) and an ABI Prism 7500 sequence detection system (Applied Biosystems, Foster City, CA, USA). Gene expression was normalized by the endogenous control of GAPDH. Each sample was separately experimented in triplicate. The primer sequences used in this study were as follows: GAPDH: forward, 5′-AGGAGCGAGACCCCACTAACA-3′; reverse, 5′-AGGGGGGCTAAGCAGTTGGT-3′; Rspo1: forward, 5′-TGTGAAATGAGCGAGTGGTC-3′; reverse, 5′-TTTGGTGTCGGAGCAGGT-3′; Rspo2: forward, 5′-AGCGAATGGGGAACGTGTAG-3′; reverse, 5′-CTTGCATCTCCTGGACTCCG-3′; Lgr4: forward, 5′-AAGATAACAGCCCCCAAGACC-3′; reverse, 5′-GCGACCAGGAAAATGAACCA-3′; Col-1a1:forward, 5′-AAGAAGCACGTCTGGTTGGAG-3′; reverse, 5′-GGTCCATGTAGGCTACGCTGTT-3′; Ocn: forward, 5′-GACCATCTTTCTGCTCACTCTGC-3′; ALP: forward, 5′-TCGGGACTGGTACTCGGATAAC-3′; reverse, 5′-GTTCAGTGCGGTTCCAGACATAG-3′; Axin2: forward, 5′-ATGATTCCATGTCCATGACG-3′; reverse, 5′-CTTCACACTGCGATGCATTT-3′; Tcf1: forward, 5′-TGCTGTCTATATCCGCAGGAAG-3′; reverse, 5′-CGATCTCTCTGGATTTTATTCTCT-3′.

### 4.10. ALP Activity Assay

BMSCs under osteogenic induction were harvested and lysed in the RIPA lysis buffer. After centrifugation, the supernatants were collected and added to 96-well plates. The ALP activity assay was performed using ALP assay kit (Beyotime Biotechnology, China) by the *p*-nitrophenyl phosphate (pNPP) method according to the manufacture’s protocol. After incubated at 37 °C for 30 min, the absorbance was detected under 405 nm using a microplate reader (Bio-Tek, Hercules, CA, USA). ALP level was normalized to the total protein content determined by the BCA protein kit (Beyotime Biotechnology, China). Finally, the ALP activity was presented as fold changes over the corresponding control group in each experiment. Every experiment was performed in triplicate independently.

### 4.11. ALP and Alizarin Red Staining

BMSCs under osteogenic induction were rinsed with PBS for three times and fixed with 4% paraformaldehyde for 30 min. At room temperature in dark, ALP staining was performed by using the BCIP/NBT regents kit (Leagene Biotechnology, Beijing, China) for 30 min, and Alizarin staining was performed by using the Alizarin working solution (Cyagen Biosciences, Shanghai, China) for 3–5 min according to the manufacturers’ instructions. After washing 3 times, the stained cells in each well were photographed immediately. Each staining experiment was repeated three times separately.

### 4.12. ELISA

The amount of Rspo1 and Ocn in cell supernatants was respectively detected by using mouse ELISA kits (Rspo1 SEA171Mu; Rspo2 SEA172Mu; Ocn SEA471Mu; Cloud-Clone Corp., Houston, TX, USA) according to the manufacturer’s instructions. All of the samples were measured in duplicate.

### 4.13. Micro-Computed Tomography Scanning and Quantitative Analysis

After being fixed overnight in 70% ethanol, the femurs were scanned by Scanco μCT 40 (Scanco Medical AG, Zurich, Switzerland) at a resolution of 18-μm. After scanning, three-/two-dimensional images were obtained and analyzed to assess bone mass, bone mineral density (BMD), bone volume over total volume (BV/TV), trabecular thickness (Tb.Th), trabecular number (Tb.N) and trabecular separation (Tb.Sp) of the distal femur. We set the region of interest (ROI) as the trabecular bone of a 2-mm length below the epiphyseal growth plate.

### 4.14. Bone Immunohistochemistry

The dissected femurs were fixed in 10% buffered formalin for 48 h, then decalcified in 10% EDTA (pH = 7.0) at 4 °C for 14–21 days, embedded in paraffin and sectioned for staining. For immunohistochemistry, distal femur sections were incubated with primary rabbit polyclonal anti-OPG (1:500 dilution) at 4 °C overnight. A horseradish peroxidase-streptavidin detection system (Dako, Glostrup, Sweden) was used to detect the immunoreactivity. Then, nuclei were counterstained with hematoxylin (Dako, Glostrup, Sweden). The sections without incubation with primary antibody were used as negative controls.

### 4.15. Skeleton Histomorphometric Analysis

For static histomorphometric analysis, the sections were subjected to Trap staining and anti-OPG immunohistochemistry staining according to the manufacturers’ protocols. To assess new bone formation, we intraperitoneally injected the mice with tetracycline (30 mg/kg, Sigma-Aldrich, St. Louis, MO, USA) and Alizarin red S (50 mg/kg, Sigma-Aldrich) on 14 days and 2 days before euthanasia. After fixation in 70% ethanol overnight, the distal femurs were embedded in methyl methacrylate and sectioned for photograph taking. Static parameters of osteoblast and osteoclast (Ob.S/BS, Ob.N/B.Pm, Oc.S/BS, and Oc.N/B.Pm) and dynamic parameters of new bone formation (mineral apposition rate (MAR) and bone formation rate (BFR)) were analyzed by using professional image analysis software (IPP 6.0) under a fluorescence microscope (Leica image analysis system, Q500MC, Heidelberg, Germany). BFR = MAR × MS (mineral surface)/BS (bone surface).

### 4.16. Statistical Analysis

Data are presented as the means ± SD of three independent experiments and quantified relative to controls (*n* is the number of tissue preparations, cells or experimental replicates). The difference among multiple groups was analyzed by ANOVA and post hoc analysis, and the difference between two groups was analyzed by Student’s *t*-test using GraphPad Prism software (Version 6.0, La Jolla, CA, USA). A difference was considered statistically significant when *p* < 0.05.

## 5. Conclusions

In conclusion, our data revealed Rspo1 as a new mechanosensitive protein in the upstream of the Wnt/β-catenin signaling pathway and highlighted the potential role of the Rspo1 and Rspo1/Lgr4 signal in bone mechanotransduction. We not only provided new insight into the mechanism of unloading induced bone loss, but also indicated that Rspo1/Lgr4 could be a potential therapeutic target for treating disuse osteoporosis. However, is there a balance between Rspo1 and sclerostin secretion in bone under mechanical stimulation? If there is, how is the balance held in the bone under the complex and dynamic mechanic circumstances for bone homeostasis? These questions need to be further investigated in future research.
